# Phenolic Compounds and Bioaccessibility Thereof in Functional Pasta

**DOI:** 10.3390/antiox9040343

**Published:** 2020-04-22

**Authors:** Valentina Melini, Francesca Melini, Rita Acquistucci

**Affiliations:** CREA Research Centre for Food and Nutrition, Via Ardeatina 546, I-00178 Roma, Italy; francesca.melini@crea.gov.it (F.M.); rita.acquistucci@crea.gov.it (R.A.)

**Keywords:** phenolic compounds, bioactive compounds, functional pasta, gluten-free pasta, bioaccessibility, bioavailability, whole grain, composite flour, legumes, food by-products

## Abstract

Consumption of food products rich in phenolic compounds has been associated to reduced risk of chronic disease onset. Daily consumed cereal-based products, such as bread and pasta, are not carriers of phenolic compounds, since they are produced with refined flour or semolina. Novel formulations of pasta have been thus proposed, in order to obtain functional products contributing to the increase in phenolic compound dietary intake. This paper aims to review the strategies used so far to formulate functional pasta, both gluten-containing and gluten-free, and compare their effect on phenolic compound content, and bioaccessibility and bioavailability thereof. It emerged that whole grain, legume and composite flours are the main substituents of durum wheat semolina in the formulation of functional pasta. Plant by-products from industrial food wastes have been also used as functional ingredients. In addition, pre-processing technologies on raw materials such as sprouting, or the modulation of extrusion/extrusion-cooking conditions, are valuable approaches to increase phenolic content in pasta. Few studies on phenolic compound bioaccessibility and bioavailability in pasta have been performed so far; however, they contribute to evaluating the usefulness of strategies used in the formulation of functional pasta.

## 1. Introduction

Phenolic compounds are secondary plant metabolites with strong antioxidant activity [1]. The consumption of food products rich in phenolic compounds has been associated with a reduced risk of chronic disease onset and ageing [2,3]. Currently, Phenol-Explorer, the first comprehensive database on polyphenol content in foods, reports the content for 500 phenolic compounds in 400 foods, for a total of 35,000 values. Fruit and vegetables are the main source of these secondary plant-metabolites.

Cereal grains contain significant amounts of phenolic compounds, as well [4,5]. Nevertheless, daily consumed cereal-based products, such as bread and pasta, are not a carrier of phenolic compounds, since they are produced with refined flour or semolina. Most bioactive compounds are concentrated in the outer layers of cereal grains which are discarded as bran, while flour and semolina are obtained from the starchy endosperm layer [6]. Hence, phenolic compounds are commonly lost during milling.

Pasta is one of the staple foods of the Mediterranean diet. It composes the base of the food pyramid and a daily consumption is recommended [7]. Pasta is a good source of carbohydrates and energy. One serving of 100 g of pasta (cooked, unenriched, without added salt) contains about 31 g of carbohydrates, 26.01 g starch, 1.8 g total dietary fibre, 5.8 g protein, and 0.93 g lipid (fat), and provides about 158 kcal [8]. When pasta is cooked al dente, it also has a low glycemic index, ranging around 32–40, depending on the pasta type [9]. Pasta glycemic index is far lower than that of bread. Additionally, pasta can possibly slow digestion rates and may contribute to longer satiety [10,11,12,13]. Pasta has also additional unquestionable advantages, such as ease of preparation, long shelf-life, low price and global consumption. It is consumed by people of all ages and from all walks of life. Hence, it may be an optimal carrier of phenolic compounds.

Currently, the focus of nutritional science has shifted toward the concept of optimal nutrition, which aims at optimizing the daily diet in terms of nutrients and non-nutrients. Hence, the demand for functional food products with a well-balanced nutritional composition and contributing to maintaining wellbeing and health, has grown.

In this framework, novel formulations of functional pasta have been proposed and innovation in pasta-making has been prompted. The aim of this paper is to identify which formulations of functional pasta contribute to a higher intake of phenolic compounds, and greater bioaccessibility and bioavailability thereof. The consumption of food products with a high number of bioactive compounds does not necessarily imply beneficial effects on human health. Bioaccessibility studies are, thus, mandatory, to evaluate the bioactivity of a functional product. To this aim, the strategies used so far in formulation of functional pasta rich in phenolic compounds, both gluten-containing and gluten-free, will be reviewed. In addition, studies on phenolic compound bioaccessibility and bioavailability in pasta will be discussed, in order to evaluate the usefulness of these strategies and provide a basis for further investigations.

## 2. Dietary Phenolic Compounds

### 2.1. Structure

Phenolic compounds are a heterogeneous group of bioactive compounds produced in plants, via either the shikimate or the acetate pathway [14]. They include a variety of chemical structures having one or more phenolic groups as a common structural feature.

Based on the number of phenol rings and the structural elements that bind rings one to another, they can be classified into: (i) simple phenols; (ii) phenolic acids; (iii) flavonoids; (iv) xanthones; (v) stilbenes; and (vi) lignans [15], while a broader classification divides phenolic compounds into flavonoids and non-flavonoids [16]. Flavonoids show a distinctive benzo-γ-pyrone skeleton and occur as aglycones, glycosides and methylated derivatives. They comprise flavonols, flavan-3-ols, flavones, isoflavones, flavanones, anthocyanidins and dihydrochalcones. Non-flavonoids include diverse classes of polyphenols, such as phenolic acids and stilbenes [16]. Among non-flavonoids of dietary significance, phenolic acids play a pivotal role and are a major class in grains. They include hydroxybenzoic acids (C6–C1), such as gallic, *p*-hydroxybenzoic, vanillic, syringic, protocatechuic and ellagic acids, as well as hydroxycinnamic acids (C6–C3), namely *p*-coumaric, caffeic, ferulic, sinapic and chlorogenic acids (Table 1).

Phenolic compounds may occur in free, soluble conjugated, and bound form, depending on whether they are bound to other constituents, or otherwise. Hence, they can be classified as free phenolic compounds (FPCs), soluble conjugated phenolic compounds (EPCs) and insoluble bound phenolic compounds (BPCs) [17]. EPCs are esterified to other molecules such as fatty acids, while BPCs are covalently bound to cell wall constituents, such as pectin, cellulose, arabinoxylans and structural proteins. BPCs are the main fraction of phenolic compounds in wheat grains [18,19].

### 2.2. Bioaccessibility, Biotransformation and Bioavailability

The concept of bioavailability in nutrition has been borrowed from pharmacology. In this discipline, the term “bioavailability” refers to the fraction of the administered dose of drug that enters systemic circulation, so as to access the site of action [20]. In nutrition, bioavailability refers to the amount of a nutrient or bioactive compound which becomes available for normal physiological functions or storage, after absorption by the gut [21].

The first step, necessary for a food component to become bioavailable, is the release from the food matrix. The extent at which a nutrient or bioactive molecule is released from the food matrix into the gastrointestinal tract and is in the right form to be absorbed, is referred to as bioaccessibility [22].

**Figure 1:**
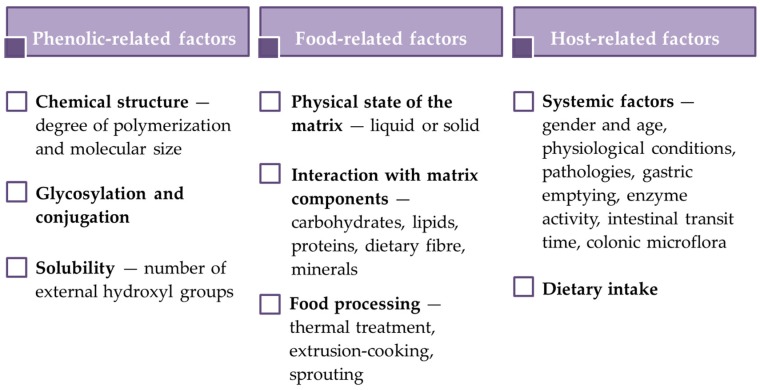
Factors affecting bioaccessibility and bioavailability of phenolic compounds.

The bioaccessibility and bioavailability of phenolic compounds are affected by factors related to phenolics, food matrix and host (Figure 1).

As regards the relationship between phenolic characteristics and bioavailability, it has been observed that chemical structure (degree of polymerization and molecular size), glycosylation and conjugation with other phenolics, and solubility are critical factors [23]. For example, phenolic acids, isoflavones, catechins and quercetin glucosides are easily absorbed, while large polyphenols are poorly absorbed.

Generally, phenolic compounds in liquid foods are more bioaccessible than those in solid foods. However, differences in phenolic bioavailability among liquid matrices have been observed. The occurrence of alcohol, dietary fibre or other nutrients, such as carbohydrates, lipids and proteins, may in fact influence phenolic compound bioavailability because of the interactions between phenolics and matrix constituents. Food processing may positively or negatively affect phenolic compound bioaccesibility and bioavailability, as well. Lafarga et al. observed that cooking increased the bioaccessibility of phenolic compounds in pulses [24]. Zeng et al. found that the content of bioaccessible phenolics in brown rice and oat significantly decreased (by 31.09% and 30.95%, respectively) after improved extrusion-cooking treatment, while in wheat they were almost unchanged, possibly because of differences in the cereal matrix [25]. It should be also considered that processing can cause a loss of phenolic compounds while promoting their bioaccessibility. Hence, bioavailability is a compromise between the compounds lost during processing and those absorbed into the organism [26].

Host-related factors—such as physiological conditions, disorders or pathologies, gastric emptying, enzyme activity, intestinal transit time and colonic microflora—may influence bioaccessibility and bioavailability of phenolic compounds, as well.

**Figure 2:**
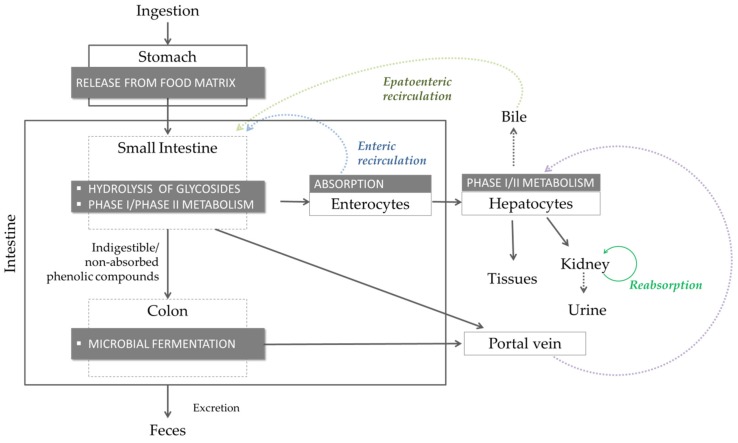
Representation of digestion, absorption and excretion of phenolic compounds and metabolites thereof.

The bioavailability of a phenolic compound implies: (i) its release from the food matrix; (ii) gastric and small-intestinal digestion (likely change of phenolic compound structure due to hydrolysis of glycosides and phase I/II metabolism); (iii) cellular uptake of aglycons and some conjugated phenolics by enterocytes; (iv) microbiological fermentation of non-absorbed polyphenols or phenolics re-excreted via bile or the pancreas, to produce additional metabolites; (v) modifications by phase I/II enzymes, upon uptake in the small intestine or in the colon; (vi) transport into the blood stream and redistribution to tissues; (vii) excretion via the kidney or re-excretion into the gut via bile and pancreatic juices (Figure 2).

Generally, after the absorption step, phenolics undergo phase I and II metabolic transformation, and metabolites with improved bioactivity or completely inactive can be obtained. As an example, protocatechuic acid, phloroglucinaldehyde, vanillic acid, and ferulic acid are bioactive metabolites obtained by the catabolism of cyanidine-3-*O*-glucoside in the gastrointestinal tract that contribute to maintaining intestinal integrity and function [27]. Hence, the evaluation of polyphenol bioavailability should include not only the determination of native compounds, but also of metabolites thereof.

#### Methods to Evaluate Phenolic Compound Bioaccessibility and Bioavailability

Several approaches have been proposed to evaluate phenolic compound bioaccessibility and bioavailability. They include the use of in vitro methods and in vivo models [23]. In vitro methods comprise simulated gastrointestinal (GI) digestion, artificial membranes, Caco-2 cell cultures and ussing chambers. As regards in vitro digestion models, they can be either static or dynamic [28]. Static models consist of multiple phases, including oral digestion (OD), gastric digestion (GD), intestinal digestion (ID) and dialysate (DIA). Each phase can vary slightly among studies. They can differ in the incubation time and characteristics of the digestive juices, and can also be adjusted for pH on the basis of the specific gut compartment [29]. However, they operate in static mode across the whole process, with prefixed conditions and parameters in terms of concentrations and volumes of digested materials, enzymes and salts, among others. The INFOGEST digestion method is an example of standardised static model [28,30].

Dynamic models include physical and mechanical processes and consider the changes that occur during the digestive process, as well as different physiological conditions. They were developed because static methods do not provide an accurate simulation of the complex dynamic physiological processes occurring under in vivo conditions. A common and very sophisticated gut model is the TIM system, a multi-compartmental dynamic computer-controlled model, used to simulate the human digestive system and to study the bioaccessibility of many compounds, such as vitamins, minerals, as well as phenolics [31].

In addition to the aforementioned methods, gastrointestinal organs in laboratory conditions (ex-vivo models) and intestinal perfusion in animals (in situ model) can be also applied in bioaccessibility/bioavailability determination [23]. In-vivo approaches are based on animal or human studies.

## 3. Strategies to Modulate Phenolic Compound Content in Pasta

According to tradition, pasta is seemingly a very simple food, produced with one ingredient, i.e., semolina of durum wheat (*Triticum turgidum* L. var. *durum*), and one “reactant”, i.e., water. Pasta final configuration, made of starch granules dispersed within the protein network, is hence obtained upon the biochemical modification of the two main constituents of durum wheat semolina (that is, proteins and starch) prompted by water addition, and mechanical and thermal energy.

Pasta by itself is a healthy food. It is a good source of carbohydrates and energy. However, in recent years, scientists and producers have been striving to develop new formulas, so that pasta can not only provide nutrients and energy, but also beneficially modulate one or more targeted functions in the body, by enhancing a certain physiological response and/or reducing the risk of disease [32]. These new formulations are known as functional pasta products.

The use of functional ingredients, such as whole grain and composite flours, as well as the addition of extracts from plant foods and food wastes, has been increasingly explored as a strategy to improve phenolic content in pasta and gluten-free pasta. In addition, processing technologies have been specifically applied to raw materials or to the pasta-making process in order to increase the content of bioactive components and their bioavailability (Table 2).

### 3.1. Use of Functional Ingredients in Pasta-Making

#### 3.1.1. Whole Grain Flours

According to the HEALTHGRAIN Consortium, whole grains (WGs) shall consist of “the intact, ground, cracked or flaked kernel after the removal of inedible parts such as the hull and husk. The principal anatomical components—the starchy endosperm, germ and bran—are present in the same relative proportions as they exist in the intact kernel” [62]. While agreement on the definition of “whole grain” has been reached, there is a lack of consensus on the definition of whole-grain foods, including “whole grain pasta” [63].

In Germany and Italy, “whole grain pasta” is pasta where 100% of the grain component in the final product is whole grain; in Denmark, pasta containing a percentage of whole grain equal or higher than 60% on a dry matter basis, can be classified as “whole grain pasta”; in France and the Netherlands, there are no regulations nor guidelines for whole grain pasta definition [63].

Two main factors explain these different levels of whole grains admitted in whole grain products. On the one hand, foods with high whole grain content are not universally appreciated by consumers, hence manufacturers need to use whole grain ingredients in a level enabling to obtain products with good sensory qualities. On the other hand, the content of whole grain ingredients used for product preparation, must be adequate to guarantee nutritional benefits to consumers.

Cereals included in the whole grain definition are wheat (including spelt, emmer, faro, einkorn, khorasan wheat, durums), rice (including pigmented varieties), barley (including hull-less or naked barley but not pearled), corn, rye, oats (including hull-less or naked oats), millets, sorghum, teff, triticale, Canary seed, Job’s tears, fonio, black fonio and Asian millet. Pseudocereals included in the whole grain definition are amaranth, buckwheat and tartar buckwheat, quinoa, and wild rice [62].

In whole grain flours, the outer multi-layered skin (bran) and the germ are retained together with the starchy main part of the grain. The bran is a major source of phenolic acids, dietary fibre (DF) and minerals, while the germ contains vitamins, minerals, fats and some proteins [64]. Phenolic acids, together with DF, are components responsible for many of the health effects associated with whole grain consumption [25]. They have shown to act synergistically and modulate favourably appetite, glucose metabolism, insulin sensitivity, and gut microbiota composition [65], and to have a role in the prevention and treatment of cardiovascular diseases [66,67]. Several studies have evidenced a lower risk from all causes and disease-specific mortality associated with a high intake of WGs [68].

The content of phenolic compounds in whole grain pasta has been, however, poorly investigated. Wójtowicz et al. determined the qualitative and quantitative profile of phenolic compounds in precooked pasta prepared from whole grain wheat and whole grain spelt [33]. Protocatechuic, 4-hydroxybenzoic, vanillic, syringic, *trans*-*p*-coumaric, *cis*-*p*-coumaric, *trans*-ferulic and *cis*-ferulic acids were identified in samples under investigation. *Cis*-ferulic acid was the main phenolic acid in both whole grain wheat and spelt pasta. In whole grain wheat pasta, vanillic acid was the second more abundant phenolic compound, while in whole grain spelt pasta, syringic and vanillic acids were identified as the main phenolics, after *cis*-ferulic acid. Compared to refined flours, the use of whole flours enabled to double the intake of phenolic acids. These data are in keeping with Chen et al. who found ferulic acid as the dominant phenolic compound in six whole grain wheat products, with values ranging between 99.9 and 316.0 µg/g [34]. In whole wheat pasta (41.4% fortification), Total Phenolic Acid (TPA) content was 226.7 µg/g dm.

Hirawan et al. determined the total phenolic content (TPC) in regular and whole grain spaghetti, and found that the former had a TPC level 2-fold lower than the latter [35]. TPC values in whole wheat spaghetti ranged between 1263 and 1423 µg/g Ferulic Acid Equivalents (FAE)/g dm, while in regular spaghetti TPC ranged between 718 and 927 µg/g FAE/g dm. It was also observed that all whole wheat spaghetti samples contained ferulic acid, while this compound was detected only in two out of regular spaghetti samples. However, TPC significantly decreased after cooking (about 40%), both in regular and whole wheat spaghetti. Despite the differences in the TPC, regular and whole grain spaghetti exhibited the same antioxidant capacity, possibly due to the antioxidant components, such as the Maillard reaction products, formed during pasta drying.

#### 3.1.2. Composite Flours

Composite flours are blends of wheat and varying proportion of legumes, tubers or other cereals, including minor cereals, and pseudocereals. Cassava, maize, rice, sorghum, millets, potato, barley, sweet potato and yam are common ingredients of composite flours [69].

The concept of using composite flours in bread and pasta-making was first elaborated to tackle a low availability of wheat in areas whose climatic conditions are not suitable for wheat production, and to encourage the use of autochthonous crops with economic advantages for local producers and consumers [69]. The concept thus first had an economic value. However, partial or total wheat substitution with composite flours affects also the nutritional profile of the final product. Wheat is, in fact, deficient in essential amino acids, such as lysine and threonine, and, during milling, bioactive compounds and minerals are commonly lost. Hence, the use of composite flours contributes to counteracting these deficiencies. More recently, the concept of “composite flours” has been thus extended to blends of wheat flour/semolina and other flours richer in essential amino acids, minerals, vitamins and phenolic compounds.

Blends of cereal flours with pulse flours have been by far explored in pasta-making. Pulses are an important source of nutrients [69]. They have a low glycemic index and are rich in complex carbohydrates, DF, plant proteins, and micronutrients. They also have high levels of polyphenols with good antioxidant properties, and other plant secondary metabolites and components (i.e., isoflavones, phytosterols, bioactive carbohydrates, alkaloids, and saponins), that are being increasingly recognized for their bioavailability and potential benefits for human health. Among phenolic compounds, phenolic acids, flavonoids and condensed tannins are the most abundant [70].

The use of pulses in pasta-making and their contribution to the content of phenolic compounds have been recently investigated by Turco et al. [36]. They found that, in pasta formulated by wheat semolina and 35% faba bean (*Vicia faba* L.) flour, TPC increased from 63.8 mg Gallic Acid Equivalents (GAE)/100 g dry matter (dm) to 185.3 mg GAE/100 g dm. Cota-Gastélum et al. prepared functional pasta with varying proportions of wheat (*T. durum* L.) semolina (0–100%), chickpea flour (0–90%), and chia flour (0–10%) [37]. In raw samples, the highest phenolic content (approximately 16 mg GAE/g) was observed when durum semolina was totally replaced and a blend of 10% chia flour and 90% chickpea flour was used. This value was approximately 8-fold higher than in durum wheat pasta (2 mg GAE/g) [37]. Carob flour, which is obtained from carob seeds, has been also used in substitution of semolina in pasta-making. Sȩczyk et al. produced pasta by using varying percentages of carob flour (1–5%) [38]. They found that the phenolic content in the produced functional pasta was higher than in the control pasta (3.51 mg GAE/g dm). In pasta with 1% of carob flour, TPC was 5.27 mg GAE/g dm, and it increased to 12.12 mg GAE/g dm in pasta with 5% carob flour [38].

Pseudocereal flours were also used to partially or totally replace semolina in pasta-making, in order to enhance pasta nutritional profile. Pseudocereals are, in fact, characterized by a high nutritional composition, in terms of high content in DF, high-quality protein, essential minerals, vitamins (e.g., folic acid), essential amino acids and unsaturated fatty acids [71,72]. They are also a valuable source of phenolic compounds [73]. Varying levels of amaranth seed flours and dried amaranth leaves (35%, 50%, 55% and 70%) were used as semolina substituents in the preparation of elbow-type pasta [39]. Both grains and leaves are, in fact, rich in bioactive compounds. Grains also show high levels of proteins (15 g/100 g) and are a source of vitamins, such as thiamine, niacin, riboflavin and folate, and minerals, namely iron, calcium, zinc, magnesium, phosphorus, copper, and manganese [74,75]. The study by Cárdenas-Hernández et al. showed that, whichever the substitution levels, amaranth pasta had a TPC higher than 100% semolina pasta (0.98 mg of FAE/g dm), with values ranging from 1.54 to 3.37 mg FAE/g dm [39]. The highest value was observed in pasta with a semolina:amaranth flour/leaves ratio of 65:35. A significant decrease in phenolic content (15–27%) was observed in all amaranth pasta samples, after cooking [39].

Composite flours have been also used to improve the nutritional value of gluten-free (GF) pasta. As a matter of fact, GF pasta is mainly produced with GF flours, such as rice and corn, which are low in micronutrients and bioactive compounds [76]. The use of blue maize in GF pasta-making has been recently explored. Blue maize (*Zea mays* L.), like the red and purple varieties, is rich in anthocyanidins (up to 325 mg/100 g dm), including cyanidin derivatives (75–90%), peonidin derivatives (15–20%) and pelargonidin derivatives (5–10%) [77]. Different percentages of blue maize (25%, 50% and 75%) were added to pasta dough produced with equal amounts of unripe plantain and chickpea flour [40]. It was observed that pasta samples containing 75% of blue maize presented the highest TPC retention after extrusion and cooking. Upon extrusion, TPC in pasta decreased between 20% and 30%, while an additional 10% loss occurred upon cooking. The phenolic compounds, retained after extrusion, were likely bound phenolics, whereas free phenolic species (e.g., free phenolic acids and anthocyanins), not physically trapped in the protein network, were leached into the cooking water.

The fortification of traditional GF flours with sorghum (*Sorghum bicolor* (L.) Moench) flour in pasta-making has been also studied. Sorghum has, in fact, high levels of a diverse array of beneficial bioactive components (e.g., polyphenols, especially flavonoids), and bioactive lipids (such as policosanols and phytosterols) [78,79,80]. Palavecino et al. produced GF pasta with white and brown sorghum [41]. They compared the two sorghum-based formulations to GF pasta produced with rice, maize and soy flour. Total phenolic compound content was higher in the two sorghum-based pasta samples than in the controls, with a value of 2.41 g GAE kg^−1^ and 2.88 g GAE kg^−1^ for white and brown sorghum, respectively. Sorghum pasta, after cooking, also showed higher radical scavenging activity and ferric reducing ability than the control samples, without significant differences between sorghum varieties.

#### 3.1.3. Powders and Extracts from Plant Foods and Food By-Products

The use of powders and extracts from plant foods and food by-products in pasta-making is among the strategies recently explored to obtain functional pasta, both gluten-containing and gluten-free.

Functional pasta was prepared by incorporating carrot powder (10%), mango peel powder (5%), moringa leaves powder (3%) and defatted soy flour (15%) in a blend of wheat semolina and pearl-millet [42]. Total flavonoid content (TFC) was determined in order to evaluate the contribution of these ingredients to the phenolic content in pasta. It emerged that, in the control pasta, TFC was 6.30 mg/100 g. The addition of mango peel powder and moringa leaves powder provided the highest values (16.53 and 17.98 mg/100 g, respectively), while carrot powder and defatted soy flour contributed at a lower extent, with values of 7.63 and 8.03 mg/100 g, respectively.

Mushrooms can also contribute to the phenolic dietary intake. The study by Lu et al. investigated the contribution of mushroom powder addition to the phenolic content of spaghetti [43]. Three different powders were used: from white button, from shiitake and from porcini mushrooms. Three different semolina substitution levels were tested: 5 g, 10 g and 15 g/100 g (*w*/*w*). It emerged that all mushroom-powder-supplemented pasta samples showed TPC values significantly higher than semolina pasta, except for 5% and 10% shiitake mushroom pasta. The greatest values were found in porcini mushroom pasta samples (approximately 4–5 mg GAE/g dm), followed by the second button mushroom samples (approximately 2 mg GAE/g dm), and shiitake mushroom pasta.

Plant food industrial processing produces huge amounts of by-products that are a serious disposal issue. However, some by-products have shown to be an abundant source of valuable compounds [81]. Hence, in the domain of circular economy, they have been increasingly turned into functional ingredients. Vegetable wastes, such as peelings, trimmings, stems, seeds, shells, and bran are some by-products from which phenolic compounds can be extracted [82]. Ultrasound-assisted extraction, microwave-assisted extraction, supercritical fluid extraction, pressurized fluid extraction, pulsed electric field extraction and enzyme-assisted extraction are green technologies commonly used in the recovery of phenolic compounds from food wastes [82,83,84]. The choice of the extraction technique is related to factors including the functional ingredient to extract and the characteristics of the food matrix. 

Onion dry skin powder has been used as a functional ingredient to modulate phenolic compound content in pasta [44]. Onion dry skins are by-products generated during industrial peeling and contain bioavailable compounds such as DF, fructo-oligosaccharides and quercetin aglycones. In the study by Michalak-Majewska et al., semolina was replaced by varying amounts of onion powder: 0%, 2.5%, 5% and 7.5%. TPC and TFC were determined both in raw and cooked samples. It was observed that pasta added with onion skin powder showed TPC and TFC higher than the control (100% semolina pasta). The highest TPC was found in pasta with 7.5% substitution level. Moreover, cooked pasta showed TPC not significantly different from the corresponding raw sample, whichever addition level of onion skin powder. Conversely, in the control pasta, TPC decreased after cooking. Hence, the functional pasta ensured a higher intake of phenolic compounds, compared to 100% semolina pasta. As regards TFC, the addition of onion skin powder enabled to obtain pasta with higher level of flavonoids, and after cooking a significant increase was observed.

Durum spaghetti were formulated by the addition of olive paste powder [45]. Olive paste is an industrial by-product of olive oil production, rich in phenolic compounds [45]. Two levels of olive paste powder were added to semolina: 10% and 15%. Phenolic content was determined on spaghetti with 10% addition of olive paste powder, since they showed the best sensory properties. TPC was 82.39 µg/g dm in the control pasta and 245.08 µg/g dm in the enriched spaghetti. In 100% semolina pasta, vanillic acid was the most abundant phenolic compound in free form (0.56 µg/g dm), while ferulic acid was the main bound phenolic compound (67.70 µg/g dm). In spaghetti enriched with olive paste powder, vanillic acid was the main phenolic acid in free form as in the control pasta; however, its content was higher (7.28 µg/g dm) than in the control. HPLC analysis also showed that the addition of olive paste powder increased the content of flavonoids, such as quercetin and luteolin.

Functional spaghetti were also produced by addition of extracts from grape marc, made up of skins, seeds, and stalks [46]. TPC was determined on fresh extruded spaghetti, pasteurized extruded spaghetti and dry spaghetti. It was found that, compared to the control, the addition of grape marc extract increased TPC in all enriched spaghetti samples (approximately 700 mg GAE/100g dm). The pasteurization and drying process did not significantly affect the TPC. Interestingly, after cooking an increase in TPC was observed, with respect to the raw samples.

Bran is the main by-product of cereal milling and is a great source of phenolic compounds and minerals. Despite its functionality, its use in pasta-making is challenging, since it has adverse effects on the quality of the final products, such as an increase of cooking loss, swelling index, and water absorption in pasta [85]. Recently, bran aqueous extract was used in the production of spaghetti [47]. The extract was obtained by ultrasound assisted-extraction at 20 °C for 25 min. The ratio between water and bran was 10 L/kg. The bran aqueous extract completely substituted processing water in pasta-making. A significant increase in phenolic compounds was observed in pasta samples due to the bran extract. In detail, TPC was 127 mg FAE/100 g fresh weight (fw) in functional spaghetti and 97 mg FAE/100 g fw in the control pasta.

As regards the formulation of functional GF pasta, different percentages (5% and 10%) of chia (*Salvia hispanica* L.) milled seeds were incorporated into rice flour dough [48]. Chia seed addition allowed increasing phenolic acid content, besides the slowly digestible starch fraction of rice, and protein and DF content. The highest content of TPAs was observed in raw samples of pasta produced with 10% milled chia seeds (164.3 µg/g). TPA content in functional GF pasta did not significantly differ from durum wheat pasta (164.3 vs 149.08 µg/g), but it was by far higher than in pasta produced with commercial GF flour (10.30 µg/g) [48]. 

After cooking, TPA content was higher in all pasta samples, with an increase of 5.3% in durum wheat pasta, 14.8% in commercial GF pasta, 25.5% in pasta with 5% of milled chia seeds and 13.7% in pasta with 10% of milled chia seeds. The highest content in TPAs was observed in pasta with a 10% milled chia seeds (186.80 µg/g). The increase in TPA content in cooked samples was possibly due to the increased bioaccessibility of bound phenolic acids after boiling [48]. Samples also differed for the content of specific phenolic acids. Addition of milled chia seeds allowed obtaining pasta samples containing chlorogenic acid, which is otherwise absent in commercial GF and durum wheat pasta. Chia seed pasta was also rich in caffeic and vanillic acids, in contrast to durum wheat pasta. The higher was milled chia seed addition, the higher was the content of chlorogenic, caffeic, and vanillic acids.

Oniszczuk et al. investigated the phenolic profile of GF pasta prepared with a blend of rice and field bean flour, enriched with different amounts (2.5%, 5%, 7.5%, 10%, 12.5% and 15%) of pear prickly fruit (*Opuntia ficus indica* (L.) Mill.) [49]. The latter is a source of phenolic compounds and also provides vitamins (C, B1, B2, A, and E), minerals (calcium, potassium, magnesium, iron, and phosphorus), and other bioactive compounds, such as carotenoids and betalains. High-performance liquid chromatography/electrospray ionization tandem mass spectrometry (HPLC-ESI-MS/MS) showed that pasta samples enriched with the different amounts of pear prickly fruit were rich in several phenolic acids: protocatechuic, caffeic, syryngic, 4-OH-benzoic, vanilic, gentisic, *trans*-sinapic, *cis*-sinapic, *p*-coumaric, ferulic, isoferulic, *m*-coumaric, 3,4-dimetoxycinnamic, and salicylic acids. The dominant acid was isoferulic. The higher was the addition of pear prickly fruit, the higher was the content of phenolic acids. Antioxidant activity was also positively correlated with the addition of fruit.

The effect of chestnut fruit (*Castanea sativa* Mill.) addition (10%, 20%, 30%, 40%, and 50%) to the aforesaid blend of rice and field bean flour on pasta phenolic content, was also investigated [50]. Chestnut fruit is rich in phenolic compounds, as well as in proteins, unsaturated fatty acids, DF, vitamins and micronutrients. As regards the content of phenolic compounds, it was observed that the total content of free phenolic acids increased along with the chestnut addition. TPA content was 38.93, 46.98, 51.47, 56.59, and 65.01 µg/g dm in samples with 10%, 20%, 30%, 40% and 50% of chestnut flour, respectively [50]. The content of each phenolic acid also increased at a higher addition of chestnut fruit, with the exception of 4-hydroxy-benzoic and salicylic acids whose level decreased at the increase of chestnut flour addition. This trend might be explained by a content of these two acids higher in the rice and field bean flour blend than in chestnut fruit powder.

### 3.2. Raw Material Processing, Pasta-Making and Pasta Cooking

In addition to the use of raw materials naturally rich in phenolic compounds, such as whole grain flour, composite flours, and plant powders and extracts, raw material processing and modulation of pasta-making and pasta cooking parameters have been explored to increase the content of phenolic compounds in pasta.

Debranning, also known as pearling, is a technology based on the gradual removal of the outer bran layers prior to milling process. While in conventional milling the aleurone layer remains attached to the bran, in debranning it remains attached to the endosperm. As a consequence, semolina and flour obtained by debranning are richer in components commonly found in the grain aleurone. The technology also enables to isolate aleurone-rich fractions, which can be used as functional ingredients [86]. Abbasi et al. have recently formulated pasta enriched with a debranning fraction from purple wheat [51]. The debranning fraction (25%) was added to flour and to semolina by dry mixing, and macaroni pasta samples were prepared. Experimental analyses on raw materials showed that phenolic compounds in wheat flour and semolina were negligible compared to the debranning fraction. Despite the debranning technology enabled to obtain raw materials rich in phenolic compounds, pasta samples showed TPC lower than it was expected. This was possibly due to the degradation of phenolics during the pasta-making process, especially in the drying step.

One more study on the formulation of pasta products by using debranning fractions was reported by Zanoletti et al. [52]. Two functional pasta products enriched with a fraction obtained from either the first or the second debranning step of purple wheat were produced. The first fraction corresponded to a debranning level of 3.7% of whole grain, while the second fraction corresponded to 6% of the debranned grain after the first step. The content of anthocyanins, a subclass of phenolic compounds typical of many fruits, vegetables, and cereal grains with red, violet, and blue colour, was determined. The analysis of cooked samples showed that anthocyanin content was 67.9 µg/g dm in pasta enriched with the first debranning fraction and 60.0 µg/g dm in pasta added with the second debranning fraction. These two values were not significantly different. Moreover, anthocyanin content in functional pasta was higher than in pasta added with bran (28 µg/g dm) and used as control sample. In addition, both functional pasta products exhibited an antioxidant activity higher than the control.

Ciccoritti et al. prepared spaghetti enriched with debranning fractions of durum wheat cv Normanno. The addition level of debranning fraction was 30% *w*/*w* [53]. Phenolic acids (PAs) and TPC were determined in raw and cooked samples, in free, esterified and bound forms. It emerged that in raw samples, free PAs content was higher in the control pasta than in functional pasta. As far as conjugated and bound PAs are concerned, values were higher in enriched samples. Conjugated PAs were 59.4 mg/kg dm and bound PAs were 650.0 mg/kg dm in functional pasta, while in control pasta they were 21.6 and 27.2 mg/kg dm, respectively. A similar trend was observed for conjugated and bound TPC. The former was 110.7 mg/kg dm in functional pasta and 31.4 mg/kg dm in control pasta, while the latter was 1308.4 and 156.9 mg/kg dm, respectively. After cooking, it was observed a higher level of PAs, whichever form was considered. Conversely, free and conjugated TPC decreased, and bound TPC increased.

Ciccoritti et al. also explored the use of micronized fractions in pasta-making [53]. Micronization is a mechanical treatment consisting in reducing kernels into a fine powder. For this reason it is also known as ultrafine grinding. It differs from conventional milling because it produces wholegrain flour, without producing by-products such as bran. The treatment damages the fibre matrix, hence the phenolic compounds linked or embedded into the matrix are more bioaccessible. The content of PAs and total phenolics in experimental pasta was determined. Raw pasta prepared from debranned and micronized durum wheat had a higher level of PAs (conjugated and bound) and total phenolics (TPs) than the control. Conjugated PAs were 36.8 mg/kg dm and bound PAs were 357.3 mg/kg dm. The content of conjugated TPs was 75.8 mg/kg dm and bound TPs were 113.3 mg/kg dm. After cooking, the level of free PAs and conjugated PAs increased, while bound PAs decreased. As regards TPs, the content of free forms did not significantly differ from raw samples, while the content of conjugated TPs decreased and the level of bound TPs increased significantly. Data are in keeping with Martini et al. who observed that micronization preserved the content of phenolic acids, while conventional milling determined 89% decrease from seeds to cooked durum wheat pasta [54].

In addition to mechanical treatments, biological processes, such as germination and fermentation, are strategies enabling to increase the phenolic compound content in pasta products.

As regards germination, both cereal grains and pulses can be sprouted. Cereal seed germination may impact on nutritional properties of cereals [87] and possibly cereal-based products. An increase in phenolic compound content ranging from 1.2 to 3.6 folds was reported in wheat, barley, sorghum, rye, oat and brown rice, after germination [87,88,89,90,91,92,93,94]. During sprouting, cell wall-degrading enzymes, such as cellulases, endoxylanases and esterases, are biosynthesized. They can hydrolyze phenolic compounds bound to cell wall constituents, so as to increase the content of free phenolic compounds. Moreover, thanks to the effect of enzymes, bound phenolic compounds are more soluble in extraction solvents and more bioaccessible. Merendino et al. explored the use of sprouted cereals in pasta-making [55]. Spaghetti were formulated by replacing wheat semolina with 30% dry tartary buckwheat sprouts belonging to the Slovenian landrace Ljse. TPC in raw tartary buckwheat spaghetti was 3.7 mg GAE/g, while it was 0.3 mg GAE/g in 100% semolina spaghetti. After cooking, TPC was 2.2 and 0.2 mg GAE/g in tartary buckwheat and in control spaghetti, respectively. Flours from sprouted legumes have been also used in pasta-making, since sprouting increases phenolic compound content in legumes [95]. In detail, Bruno et al. investigated the contribution of sprouting to increasing phenolic content in chickpea pasta [56]. Pasta prepared with sprouted chickpea flour had phenolic content 15% higher than non-sprouted chickpea pasta.

Sourdough-fermented ingredients have been recently proposed to enhance the nutritional and functional properties of pasta [96]. However, to our knowledge, no study investigating the phenolic profile of pasta produced with sourdough-fermented flours has been so far published. Fermentation is indeed one more pre-processing technique that can increase the content of phenolic compounds in pasta ingredients, such as pulses and pseudocereals, and bran. Microorganisms responsible for the fermenting process, produce enzymes which boost the release of insoluble bound phenolic compounds from the food matrix, thus increasing their solubility and bioaccessibility. Rashid et al. reported a content of extractable phenolic compounds, in rice bran fermented by *Aspergillus oryzae*, 3.8-fold higher than in unfermented bran [97]. It was also observed that rice bran solid-state fermentation with *A. oryzae* affected the profile of phenolic acids. In unfermented bran, protocatechuic, coumaric and ferulic acids were found, while in fermented rice bran *p*-coumaric, protocatechuic, ferulic, caffeic and sinapic acids were detected. Moreover, the level of coumaric, ferulic and protocatechuic acids in the fermented bran increased by up to 3.2-fold, 52-fold and 3.2-fold, respectively, compared to its unfermented counterpart. Dey et al. studied the effect of solid state fermentation of wheat by *Rhizopus oryzae* RCK2012 on phenolic content and they found that TPC increased from 5.15 mg GAE/g to 24.55 mg GAE/g [98]. Călinoiu et al. explored the use of solid-state fermentation to improve phenolic content in wheat and oat bran [99]. A 112% increase in TPC was observed on day 3 fermentation in wheat bran and 83% increase on day 4 of fermentation in oat bran, with values reaching 0.84 mg GAE/g and 0.45 mg GAE/g, respectively. Based on these results, it can thus be speculated that fermentation of raw materials may contribute to increasing phenolic content in pasta; however, additional studies on the effect of pasta-making upon TPC are required.

The pasta-making process can also influence the content of phenolic compounds; process parameters and conditions may be thus set in order to limit/avoid phenolic compound degradation and/or increase their bioaccessibility.

Generally speaking, conventional pasta is produced by forcing flour/semolina dough through a die to obtain the required shape, and then drying it. Low shear and heat values (30–40 °C) are applied. Gluten-free and precooked pasta are prepared by extrusion-cooking, which is a high-temperature short-time process consisting in a short-term heating of dough, at a high temperature, under high pressure. During extrusion-cooking, raw materials are forced to flow through a die and thermal and shear energies cause structural, chemical, and nutritional transformations, including gelatinization and degradation of starch, denaturalization of proteins, oxidation of lipids, degradation of vitamins and bioactive compounds, and changes in bioavailability of minerals and solubility of dietary fibre [100]. The combination of high temperature, high pressure, and high shearing conditions during extrusion-cooking may affect the content in phenolic compounds. Heat can cause the decomposition of heat-labile phenolics and polymerization of some phenolic compounds, thus decreasing their content. At the same time, heat disrupts cell wall matrices which hinder phenolic molecules to gastrointestinal enzymes, thus promoting their accessibility [101]. Hence, the effect of extrusion-cooking on phenolic content and bioaccessibility depends on which effect prevails.

As regards the effect of extrusion-cooking parameters on phenolic content in pasta, Bouasla et al. observed that the application of higher screw speed (80 rpm) enabled to obtain higher phenolic content in GF precooked rice-yellow pea pasta [57]. As a matter of fact, in cereal grains phenolic acids are mainly found in the bound form and such complexes are difficult to break down at lower screw speeds [57]. Oniszczuk et al. studied the effect of extruder screw speed on free phenolic acid content of GF precooked pasta obtained from roasted buckwheat (*Fagopyrum esculentum* Moench and *F. tataricum* Gaertner) flour [58]. The qualitative and quantitative analysis of FAs in extruded pasta by high-performance liquid chromatography electrospray ionization tandem mass spectrometry (HPLC-ESI-MS/MS) showed that gallic, protocatechuic, gentisic, 4-hydroxybenzoic, vanilic, *trans*-caffeic, *cis*-caffeic, *trans*-*p*-coumaric, *cis*-*p*-coumaric, syryngic, *trans*-ferulic, *cis*-ferulic, salicylic, *trans*-sinapic and *cis*-sinapic acids were present in all pasta samples, regardless of moisture content (30%, 32% and 34%) and screw speed (60, 80, 100 and 120 rpm). However, in pasta samples produced at 100 rpm extruder screw speed and 32% flour moisture content, benzoic acid derivatives (i.e., gallic, protocatechuic, gentisic, 4-hydroxybenzoic and salicylic acids) were present with the highest amounts. The highest content in cinnamic acid derivatives (i.e., *trans*-caffeic, *trans*-*p*-coumaric, *cis*-*p*-coumaric and *cis*-ferulic) was observed in samples of GF buckwheat pasta produced at 60 rpm extruder screw speed and 30% of flour moisture [58]. Conversely, De Paula et al. reported a significant reduction in total phenolic acid content after pasta extrusion, possibly due to oxidising reactions promoted by water, oxygen and heat [59].

Cooking is a necessary step for pasta consumption, and it may influence the content of phenolic compounds and/or change the ratio between free and bound form of phenolics. De Paula et al. investigated the effect of cooking on phenolic content in barley pasta and observed that TPAs were not greatly affected by this treatment, and both free and bound phenolic compounds were preserved [59]. Conversely, Podio et al. found that cooking promotes the release of bound phenolic compounds, thus increasing the content of the free forms. In addition, pasta-making and cooking produced a change in the phenolic profile with respect to the starting flour [60]. Results are in keeping with Rocchetti et al. who observed that cooking by boiling lowered the bound-to-free ratio of phenolics in GF pasta [61]. They studied six commercially available GF pasta samples (i.e., pasta enriched with black rice, chickpea, red lentil, sorghum, amaranth and quinoa) and observed that in raw GF pasta samples, bound TPC was higher than free TPC, with values ranging from 7.58 mg GAE/100 g (sorghum GF pasta) to 32.68 mg GAE/100 g (quinoa GF pasta). After cooking, the highest free TPC was observed in black rice and quinoa samples, with 27.27 and 19.27 mg GAE 100 g^−1^, respectively (*p* < 0.01). In conclusion, from a nutritional point of view, understanding the effect of processing on phenolic content and on the ratio between free and bound forms is pivotal. As a matter of fact, the activity of phenolic acids is strictly dependent on the form they reach the gastrointestinal tract. The intake of free forms or soluble conjugated forms has systemic beneficial effects, such as inhibition of LDL cholesterol and liposome oxidation, since they are rapidly absorbed in the stomach and small intestine. Conversely, insoluble bound phenolic compounds reach the colon nearly intact where they are hydrolysed by the esterases and xylanase of colon microorganisms, thus having local activity and protecting against colon cancer [102]. However, the effects of phenolic compounds on human health depend on both the amount consumed and the bioavailability thereof.

## 4. Bioaccessibility of Phenolic Compounds in Pasta

As discussed above, several strategies have been explored in order to increase the phenolic content in pasta. Thanks to its low cost and long shelf life, pasta is consumed by people of all ages and all walks of life, hence it is appropriate to be used as a carrier of phenolic compounds, in order to promote health and wellbeing. However, a high dietary phenolic compound intake does not necessarily imply an appropriate bioactivity. As a matter of fact, bioactivity strictly depends on phenolic compound bioaccessibility and bioavailability. Several studies have been published and reviewed on the bioaccessibility of phenolic compounds in bread [29], while bioaccessibility of phenolic compounds in pasta products has been poorly investigated.

Phenolic compound bioaccessibility has been studied in pasta products formulated with whole-wheat flour or composite flours and in pasta samples produced with powders from plant materials or food by-products. Polyphenol bioaccessibility in GF pasta has been investigated, as well (Table 3). Static digestion models have been mainly used.

Podio et al. investigated phenolic compound bioaccessibility in whole-wheat pasta by using an experimental model, simulating human gastrointestinal digestion and subsequent absorption [60]. They observed that the conditions found in the intestinal medium (e.g., alkaline pH, pancreatin and bile actions, etc.) are not favourable for the stability of some phenolic compounds, which are changed, among others, by enzymatic, oxidative and other transformations, and by aggregation with food matrix. Generally speaking, they observed that TPC significantly increased after gastric digestion (GD) and intestinal digestion (ID), but in the dialysate (DIA) it was significantly lower than in the GD and ID. As to the polyphenol profile, only 8 out of the 25 compounds identified and quantified in cooked pasta were detected in the four stages of the in vitro digestion. In particular, the analysis of dialysated samples showed that hydroxybenzoic acid diglucoside, hydroxybenzoic acid glucoside, tryptophan and *trans*-ferulic acid content increased with respect to the corresponding intestinal digestion. This is of paramount importance as these compounds represent the bioaccessible and dialyzable fraction of polyphenols, which pass into the blood stream to reach organs or tissues where they would exert their antioxidant action. The authors hypothesized that the alkaline conditions and the action of pancreatin/porcine bile acting during the intestinal phase boosted the release of these phenolic compounds from dietary fibre.

Pigni et al. performed a simulated in vitro gastrointestinal digestion of cooked samples of wheat pasta fortified with 10% of partially-deoiled chia flour (PDCF), to assess the absorption of individual polyphenols through the different stages [103]. Upon oral digestion (OD), a total of 50% of the TPC found in the cooked supplemented pasta was released. Gastric digestion and intestinal digestion determined a higher increase (i.e., 300–500%) indicating that the action of enzymes (pepsin, pancreatin) and pH enables an effective release of polyphenols from the food matrix, including the components of PDCF and wheat. Finally, the DIA samples, representing the fraction absorbed in the intestine, showed an increase of around 50% compared with the values of boiled pasta. As regards the specific phenolic compounds quantified in boiled pasta, only 2 out of 10 were above the limit of detection (LOD) and limit of quantitation (LOQ) in the intestinal samples of pasta with 10% PDCF: rosmarinic acid and salviaflaside. In the DIA samples they were even below the LOQ, however their detection indicates that at least a small fraction is being absorbed at this stage.

Marinelli et al. investigated the bioaccessibility of phenolic compounds in samples of pasta produced with durum wheat semolina and red grape marc, a by-product of winemaking, in combination with transglutaminase [104]. They found that the functional pasta sample showed a significantly higher concentration of bioaccessible total polyphenols than the control sample, formulated only with durum wheat semolina (5.53 vs 4.16 mg GAE/g dm, respectively).

Another study investigated the bioaccessibility and potential bioavailability of phenolics in pasta produced by substituting wheat flour (2.5% and 7.5%) with lyophilised raspberries (*Rubus idaeus* L.), boysenberries (*Rubus idaeus* × *Rubus ulmifolius*), redcurrants (*Ribes rubrum* L.) and blackcurrants (*Ribes nigrum* L.) [105]. It was observed that potentially bioaccessible polyphenols were higher in pasta enriched with fruits from *Rubus* genus than with *Ribes* fruits. Pasta fortified with raspberries and boysenberries showed an increase of 260% in polyphenols, while in samples enriched with red- and blackcurrants, the increase was 360%.

As regards the bioaccessibility of phenolics in GF pasta, Camelo-Méndez et al. investigated samples produced with flours from unripe plantain (*Musa paradisiaca* L.), chickpea and blue maize by using an in vitro model simulating gastrointestinal digestion [106]. During the oral digestion, only free polyphenols were released from the matrix, that is, those compounds not linked to other molecules, such as proteins, lipids and carbohydrates. During the gastric phase, the release of phenolic compounds was higher in samples with a higher amount of blue maize flour (i.e., 50% and 75%). The higher release was likely associated with the breakdown of complexes with proteins, fibre residues and sugars. The low pH and enzymatic activity also favour the release of phenolic compounds, mainly flavonoids, from the food matrix. After ID, the percentage of phenolic compounds released was 40% of the initial value in the samples. In detail, they observed that the bioaccessibility of the phenolic compounds in pasta was up to 80% and the highest amount was obtained with the pasta manufactured with the highest amounts of blue maize.

Palavecino et al. also studied bioaccessibility of the functional GF pasta they produced with two varieties of sorghum, and found that the white and brown sorghum pasta samples had 2.9- and 2.4-fold higher potentially bioaccessible polyphenol content than in cooked sample, respectively [41]. The antioxidant activity in sorghum pasta did not significantly vary after digestion, and it was approximately 36–48% in DIA samples.

Rocchetti et al. investigated phenolic compound bioaccessibility in six samples of commercially available pasta, formulated with black rice, chickpea, red lentil, sorghum, amaranth and quinoa [107]. They used an in vitro gastrointestinal digestion model comprising two steps: a pre-incubation step with digestive enzymes, and an in vitro large intestine fermentation process. The phenolic profile was investigated at different time points during faecal fermentation. It emerged that GF pasta samples enriched with pseudocereals or legumes were able to deliver phenolics to the large intestine, and this was likely due to the contribution of the food matrix, which acts as a carrier. In addition, once in the large intestine, the main phenolic subclasses (i.e., flavonoids, hydroxycinnamic acids, lignans and stilbenes) degraded, along with a parallel increase in low molecular weight phenolic acids (i.e., hydroxybenzoic acids), alkylphenols, hydroxybenzoketones and tyrosols. As regards phenolic compound bioaccessibility during the large intestine fermentation process, flavonoids reported values lower than 1%, regardless of the time point or matrix considered. Hydroxycinnamic acid bioaccessibility in large intestine ranged from 0.6% to 8.6% at 0 h, from 0.6% to 1.6% at 8 h, and from 0.7% to 5.5% at 24 h. Within lignans, the various classes showed differences in bioaccessibility, with furofurans having very low bioaccessibility, dibenzylbutyrolactones reached the colon in larger amounts (i.e., 2.7–12.2% of bioaccessibility); while tyrosols and alkylresorcinols were the phenolics with the highest bioaccessibility during the in vitro fermentation process.

## 5. Conclusions

Phenolic compounds have documented beneficial effects on human health, because of their contribution to preventing chronic diseases. Durum wheat semolina, the main ingredient of pasta, lacks phenolic compounds, since they are lost during conventional milling. Hence, several strategies have been proposed to produce functional pasta whose consumption may contribute to an increased intake of phenolic compounds. Whole grain, legume and composite flours are the main substituents of durum semolina. GF pasta has been functionalized, as well, by using ingredients rich in phenolic compounds. The use of pre-processing technologies on raw materials, such as sprouting, or modulation of extrusion-cooking conditions, may be valuable approaches to increase the phenolic content in pasta. However, a higher intake of phenolic compounds does not necessarily imply a greater bioactivity. Hence, it is pivotal to investigate bioaccessibility and bioavailability of phenolic compounds in functional pasta. Currently, few studies have been performed, and comparing results across different studies is not always reliable due to the diversity of in vitro model conditions and the lack of official methods for the determination of phenolic compound content. Hence, efforts are still needed to evaluate the contribution of functional pasta consumption to maintaining optimal health.

## Figures and Tables

**Table 1:** Major classes of dietary phenolic compounds, skeleton structure thereof and common representatives.

Class	Subclass	Skeleton Structure	Common Representatives
Flavonoids	Flavonols	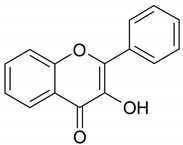	Kaempferol, quercetin
	Flavan-3-ols	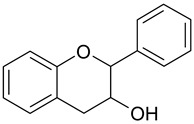	Catechin, gallocatechin, epicatechin
	Flavones	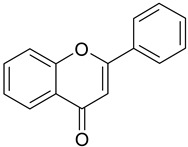	Luteolin, apingenin
	Isoflavones	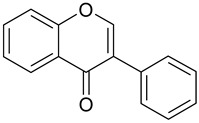	Genistein, daidzein
	Flavanones	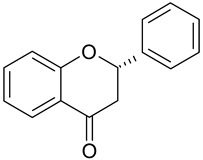	Naringenin, hesperetin
	Anthocyanidins	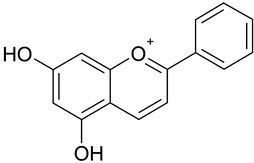	Cyanidin, malvidin, delphinidin
	Dihydrochalcones	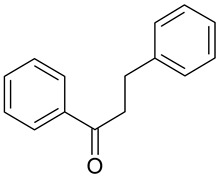	Phloretin
Non-Flavonoids	Phenolic acids—Hydroxybenzoic acids	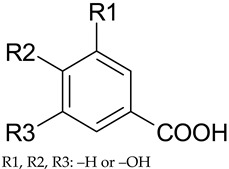	Gallic acid, *p*-hydroxybenzoic acid, vanillic acid, syringic acid, protocatechuic acid, ellagic acid
	Phenolic acids—Hydroxycinnamic acids	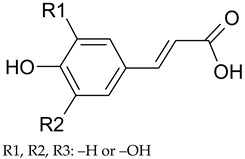	*p*-coumaric acid, caffeic acid, ferulic acid, sinapic acid, chlorogenic acid
	Stilbenes	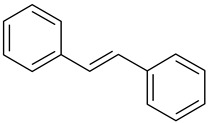	Resveratrol

**Table 2:** Modulation of phenolic compound content in pasta.

Strategy	Sub-Strategy	Pasta Products	Effect on Phenolic Compound Content/Profile	Reference
Use of functional ingredients in pasta-making	Whole Grain Flours	Whole grain wheat and whole grain spelt pasta (precooked)	↑ content of protocatechuic, 4-hydroxybenzoic, vanillic, syringic, *trans*-*p*-coumaric, *cis*-*p*-coumaric, *trans*-ferulic and *cis*-ferulic acids.	Wójtowicz et al. [33]
		Whole grain wheat products	TPAs: 226.7 µg/g	Chen et al. [34]
		Whole grain spaghetti	TPC (whole wheat spaghetti): 1263–1423 µg FAE/g dm TPC (regular spaghetti): 718–927 µg FAE/g dm	Hirawan et al. [35]
	Composite Flours	Pasta formulated with wheat semolina and 35% faba bean (*Vicia faba* L.) flour	TPC (functional pasta): 185.3 mg GAE/100 g dm TPC (control pasta): 63.8 mg GAE/100 g dm	Turco et al. [36]
		Pasta with varying proportions of wheat (*T. durum* L.) semolina (0–100%), chickpea flour (0–90%), and chia flour (0–10%)	TPC (pasta with 10:90 chia:chickpea flour ): 16 mg GAE/g dm TPC (control pasta): 2 mg GAE/g	Cota-Gastélum et al. [37]
		Pasta prepared with carob flour (1–5%)	TPC (pasta with 1% of carob flour): 5.27 mg GAE/g dm TPC (pasta with 5% carob flour): 12.12 mg GAE/g dm TPC (control pasta): 3.51 mg GAE/g dm	Sȩczyk et al. [38]
		Pasta prepared with amaranth seed flours and dried amaranth leaves (35%, 50%, 55% and 70%)	TPC (amaranth-added pasta): 1.54 to 3.37 mg FAE/g dm TPC (control pasta, 100% semolina): 0.98 mg FAE/g dm The highest value was observed in pasta with a semolina: amaranth flour/leaves ratio of 65:35.	Cárdenas-Hernández et al. [39]
		GF pasta (unripe plantain and chickpea flour ) added with blue maize (*Zea mays* L.) at 25%, 50% and 75%	Samples containing 75% of blue maize presented the highest TPC retention after extrusion and cooking (approx. 70% and 80%, respectively). In the control pasta, the phenolic retention after extrusion and cooking was approx. 52% and 60%, respectively.	Camelo-Méndez et al. [40]
		GF pasta (with rice, maize and soy flour) added with white and brown sorghum	TPC (pasta with white sorghum): 2.41 g GAE/ kg TPC (pasta with brown sorghum): 2.88 g GAE/kg TPC (rice pasta): 0.37 g GAE/kg TPC (soy pasta): 1.37 g GAE/kg TPC (corn pasta): 0.52 g GAE/kg	Palavecino et al. [41]
	Powders and extracts from plant foods and food by-products	Pasta from wheat semolina and pearl-millet added with carrot powder (10%), mango peel powder (5%), moringa leaves powder (3%) and defatted soy flour (15%)	TFC (control pasta): 6.30 mg/100 g dm TFC (carrot-added pasta): 7.63 mg/100 g dm TFC (mango peel-added pasta): 16.53 mg/100 g dm TFC (moringa leaves-added pasta): 17.98 mg/100 g dm TFC (defatted soy flour-added pasta): 8.03 mg/100 g dm	Jalgaonkar et al. [42]
		Pasta added with mushroom (white button, shiitake and porcini) powder, at 5%, 10% and 15% semolina substitution levels	TPC values in mushroom pasta were significantly higher than in control pasta, except for 5% and 10% shiitake mushroom pasta. The greatest values were found in porcini mushroom pasta samples (approximately 4–5 mg GAE/g dm).	Lu et al. [43]
		Pasta added with onion powder, at 0%, 2.5%, 5% and 7.5% semolina substitution level	TPC (cooked pasta added with onion skin): approx. from 1.4 to 3 mg GAE/g dm TFC (cooked pasta added with onion skin): approx. from 0.7 to 3.8 mg QE/g dm TPC (cooked control pasta): approx. 0.5 mg GAE/g dm TFC (cooked control pasta): approx. 0.1 mg QE/g dm Cooked pasta showed TPC not significantly different from the corresponding raw sample, whichever addition level of onion skin powder.	Michalak-Majewska et al. [44]
		Durum spaghetti added with olive paste powder (10%)	TPC (enriched spaghetti): 245.08 µg/g dm TPC (control pasta): 82.39 µg/g dm Control and functional pasta differed also in the phenolic profile. Increased level of flavonoids (i.e., quercetin and luteolin) in functional pasta.	Padalino et al. [45]
		Spaghetti added with extracts from grape marc (grape skins, seeds, and stalks)	TPC (functional spaghetti): approx. 700 mg GAE/100g dm	Marinelli et al. [46]
		Pasta prepared from semolina and bran aqueous extract	TPC (functional spaghetti): 127 mg FAE/100 g fw TPC (control pasta): 97 mg FAE/100 g fw	Pasqualone et al. [47]
		GF pasta added with chia (*Salvia hispanica* L.) milled seeds (5% and 10% substitution levels)	In raw samples— TPA (functional GF pasta—10% sub.): 164.3 µg/g TPA (durum wheat pasta): 149.08 µg/g TPA (functional GF pasta—5% sub.): 98.40 µg/g TPA (pasta produced with commercial GF flour): 10.30 µg/g In cooked samples— ↑ TPAs in all pasta samples. TPA (functional GF pasta—10% sub.): 186.80 µg/g TPA (durum wheat pasta): 156.99 µg/g TPA (functional GF pasta—5% sub.): 123.53 µg/g TPA (pasta produced with commercial GF flour): 11.83 µg/g Control and functional pasta also differed in the phenolic profile.	Menga et al. [48]
		GF pasta (from a blend of rice and field bean flour) added with pear prickly fruit (*Opuntia ficus indica* (L.) Mill.) in different amounts (2.5%, 5%, 7.5%, 10%, 12.5% and 15%)	Pasta samples enriched with pear prickly fruit were rich in several phenolic acids, namely protocatechuic, caffeic, syryngic, 4-OH-benzoic, vanilic, gentisic, *trans*-sinapic, *cis*-sinapic, *p*-coumaric, ferulic, isoferulic, *m*-coumaric, 3,4-dimetoxycinnamic, and salicylic acids. The higher was the addition of pear prickly fruit, the higher was the content of phenolic acids. The dominant acid was isoferulic.	Oniszczuk et al. [49]
		GF pasta (from a blend of rice and field bean flour) added with chestnut fruit (*Castanea sativa* Mill.) in different amounts (10%, 20%, 30%, 40%, and 50%)	TPA content (10%): 38.93 µg/g dm TPA content (20%): 46.98 µg/g dm TPA content (30%): 51.47 µg/g dm TPA content (40%): 56.59 µg/g dm TPA content (50%): 65.01 µg/g dm The content of each phenolic acid also increased at the higher addition of chestnut fruit, with the exception of 4-hydroxy-benzoic and salicylic acids.	Oniszczuk et al. [50]
Raw material processing, pasta-making and pasta cooking	Debranning	Pasta enriched with a debranning fraction from purple wheat (25%)	Phenolic compounds in wheat flour and semolina were negligible compared to the debranning fraction from purple wheat. In pasta samples TPC was lower than it was expected. This was possibly due to the degradation of phenolics during the pasta-making process.	Abbasi et al. [51]
		Pasta enriched with the first and the second debranning fraction from purple wheat	Anthocyanin content (pasta enriched with the 1st debranning fraction): 67.9 µg/g dm Anthocyanin content (pasta added with the 2nd debranning fraction): 60 µg/g dm Anthocyanin content (control pasta with bran addition): 28 µg/g dm	Zanoletti et al. [52]
		Spaghetti enriched (30%) with debranning fractions of durum wheat	In raw samples— Free PAs were higher in the control pasta than in functional pasta. Conjugated PAs (functional pasta): 59.4 mg/kg dm Conjugated PAs (control pasta): 21.6 mg/kg dm Bound PAs (functional pasta): 650.0 mg/kg dm Bound PAs (control pasta): 27.2 mg/kg dm Conjugated TPC (functional pasta): 110.7 mg/kg dm Conjugated TPC (control pasta): 31.4 mg/kg dm Bound TPC (functional pasta): 1308.4 mg/kg dm Bound TPC (control pasta): 156.9 mg/kg dm In cooked samples— ↑ level of PAs, whichever form was considered ↓ free and conjugated TPC ↑ level of bound phenolic compound	Ciccoritti et al. [53]
	Micronization	Pasta added with micronized fractions	In raw functional pasta— Conjugated PAs: 36.8 mg/kg dm Bound PAs: 357.3 mg/kg dm Conjugated TPs: 75.8 mg/kg dm Bound TPs: 113.3 mg/kg dm In cooked functional pasta (with respect to raw samples)— ↑ free PAs and conjugated PAs ↓ bound PAs ↓ conjugated TPs ↑ bound TPs	Ciccoritti et al. [53]
		Pasta added with micronized fractions	Micronization preserved the content of phenolic acids, while conventional milling determined 89% decrease from seeds to cooked durum wheat pasta	Martini et al. [54]
	Cereal germination	Spaghetti formulated by using 30% dry tartary buckwheat sprouts	In raw samples— TPC (raw tartary buckwheat spaghetti): 3.7 mg GAE/g TPC (100% semolina spaghetti): 0.3 mg GAE/g In cooked samples— TPC (raw tartary buckwheat spaghetti): 2.2 mg GAE/g TPC (100% semolina spaghetti): 0.2 mg GAE/g	Merendino et al. [55]
	Legume germination	Pasta prepared with sprouted chickpea flour	TPC (sprouted chickpea pasta): 8.4 mg GAE/g TPC (non-sprouted chickpea pasta): 7.3 mg GAE/g	Bruno et al. [56]
	Extrusion and Extrusion-cooking	GF precooked rice-yellow pea pasta	↑ TPC at higher screw speed (80 rpm)	Bouasla et al. [57]
		GF precooked pasta from roasted buckwheat (*Fagopyrum esculentum* Moench and *F. tataricum* Gaertner) flour	Highest level of benzoic acid derivatives (i.e., gallic, protocatechuic, gentisic, 4-hydroxybenzoic and salicylic acids) at 100 rpm extruder screw speed and 32% flour moisture content. Highest content in cinnamic acid derivatives (i.e., trans-caffeic, *trans*-*p*-coumaric, *cis*-*p*-coumaric and *cis*-ferulic acids) at 60 rpm extruder screw speed and 30% of flour moisture	Oniszczuk et al. [58]
		Barley pasta	↓ TPC after extrusion	De Paula et al. [59]
	Cooking	Barley pasta	TPAs were not greatly affected by cooking	De Paula et al. [59]
		Whole wheat	↑ free TPC	Podio et al. [60]
		GF pasta (i.e., pasta enriched with black rice, chickpea, red lentil, sorghum, amaranth and quinoa)	In raw GF pasta— Bound TPC > Free TPC Bound TPC (sorghum GF pasta): 7.58 mg GAE/100 g Bound TPC (quinoa GF pasta): 32.68 mg GAE/100 g In cooked GF pasta— Free TPC > Bound TPC Free TPC (black rice pasta): 27.27 mg GAE/100 g Free TPC (quinoa pasta): 19.27 mg GAE/100 g	Rocchetti et al. [61]

↓: decrease; ↑: increase; dm: dry matter; FAE: Ferulic Acid Equivalents; fw: fresh weight; GAE: Gallic Acid Equivalents; GF: Gluten-free; PAs: Phenolic Acids; QE: Quercetin Equivalents; TFC: Total Flavonoid Content; TPA(s): Total Phenolic Acid(s); TPC: Total Phenolic Content; TPs: Total Phenolics.

**Table 3:** Bioaccessibility studies on phenolic compounds in pasta.

Pasta Formulation	Phenolic Compounds Analysed	In Vitro Methods	Main Findings	Reference
Pasta produced with two varieties of whole wheat flour (*Triticum aestivum* L.)	TPC, 6G8AA, 8G6AA, *c*FA, ChDP, DFA (Isomers 1–12), FAD, HBADG, HBAG, HGPBA, *p*CoA, *p*CoFP, *t*FA, TFA	OD: human saliva, homogenization, pH adjustment to 2. GD: addition of pepsin solution (pepsin + 0.1 M HCl) to the homogenate; incubation with shaking for 2 h at 37 °C. ID and DIA: addition of a pancreatin/porcine bile solution and dialysis for 3 h at 37 °C.	After OD: release of 4.5–11% of TPC found in cooked supplemented pasta (depending on the variety). After GD: ↑ (344–370%) of TPC found in cooked supplemented pasta. After ID: ↑ (340–360%) of TPC found in cooked supplemented pasta. After DIA: ↑ (~140%) of TPC found in cooked supplemented pasta. Hydroxybenzoic acid diglucoside, hydroxybenzoic acid glucoside and *trans*-ferulic acid were the main compounds quantified in DIA samples.	Podio et al. [60]
Pasta from wheat flour fortified with partially-deoiled chia flour	QA, SA I/H, CTA, FTA, Try, CAH, CA, SA E/B/L, SF, RA, SA C, MeRA, MeQ	OD: human saliva; homogenization; pH adjustment to 2. GD: pepsin solution (pepsin + 0.1 M HCl) added to the homogenate; incubation with shaking for 2 h at 37 °C. ID and DIA: addition of a pancreatin/porcine bile solution and dialysis for 3 h at 37 °C.	After OD: release of 50% of the TPC found in cooked supplemented pasta. After GD and ID: ↑ (300–500%) of TPC found in cooked supplemented pasta. After DIA: ↑ (~50%) of TPC found in cooked supplemented pasta.	Pigni et al. [103]
Pasta produced with durum wheat semolina, red grape marc (RGM) and transglutaminase (TG)	TPC	GD: porcine pepsin; pH = 2.2–2.4; incubation with shaking for 1 h at 37 °C. ID: addition of porcine bile acid, pancreatin, α-amylase; pH = 7.2–7.6; treatment with nitrogen gas and shaking at 37 °C in a water bath for 2 h.	Bioaccessible TP in RGM/TG pasta vs control: 5.53 ± 0.61 vs. 4.16 ± 0.50 mg GAE/g dm	Marinelli et al. [104]
Pasta enriched with fruits from *Rubus and Ribes* genus	TPC	Based on the static method proposed by INFOGEST’s scientists [30]	↑ (260%) of TPC (raspberry- and boysenberry-enriched pasta). ↑ (360%) of TPC (red- and blackcurrant enriched pasta).	Bustos et al. [105]
GF pasta formulated with blue maize, chickpea and unripe plantain flours	FPCs and TPC	OD: food was chewed for 15 s; each person rinsed his/her mouth with 5 mL of phosphate buffer. GD: HCl-KCl buffer; pH = 1.25; pepsin solution; incubation at 40 °C in a water bath for 60 min. ID: addition of a mixture of enzymes, incubated for 1 h at 37 °C in a water bath with constant agitation. DIA: dialysis tubing; pancreatic α-amylase solution; incubation at 37 °C.	After OD: release of FPCs. After GD: ↑ TPC release at the increase of blue maize flour percentage. After ID: release of 40% TPC.	Camelo-Méndez et al. [106]
GF pasta produced with white and brown sorghum	TPC	OD: simulated salivary fluid as reported in [108], sample disrupted in a Teflon pestle, incubated for 2 min at 37 °C. GD: simulated stomach fluid as reported in [108]; pH adjusted to 3; incubation for 2 h at 37°C. ID: simulated duodenal fluid as reported in [108]; pH adjusted to 7; incubation for 3 h at 37°C.	Phenolic compound bioaccessibility of white and brown sorghum GF pasta was 2.9- and 2.4-fold higher than in cooked pasta, respectively.	Palavecino et al. [41]
GF pasta produced with black rice, chickpea, red lentil, sorghum, amaranth and quinoa	TPC Flavonoids Lignans Stilbenes	Pre-incubation step with digestive enzymes. In vitro large intestine fermentation process.	After the large intestine fermentation process: - Flavonoid bioaccessibility: <1% - Hydroxycinnamic acid bioaccessibility: 0.6% to 8.6% (at 0 h), 0.6% to 1.6% (at 8 h) and 0.7% to 5.5% (at 24 h) - Lignan bioaccessibility: furofurans (very low); dibenzylbutyrolactones (2.7–12.2%); tyrosols and alkylresorcinols (the most bioaccessible).	Rocchetti et al. [107]

↑: increase; 6G8AA: 6-C-glucosyl-8-C-arabinosyl-apigenin; 8G6AA: 8-C-Glucosyl-6-C-arabinosyl-apigenin; CA: Caffeic acid; CAH: Caffeic acid hexoside; ChDP: Chrysoeriol-6,8-di-C-pentoside; cFA: cis-ferulic acid; CTA: Caftaric acid; DFA (Isomers 1, 2, 3, 4, 5, 6, 7, 8, 9, 10, 11, 12): Diferulic acid; DIA: dialysate; FAD: Ferulic acid derivative; FPCs: Free Phenolic Compounds; FTA: Fertaric acid; GD: gastric digestion; HBADG: Hydroxybenzoic acid diglucoside; HBAG: Hydroxybenzoic acid glucoside; HGPBA: 2-Hydroxy-3-*O*-β-d-glucopyranosylbenzoic acid; ID: intestinal digestion; MeQ: Methylquercetin; MeRA: Methylrosmarinate; OD: oral digestion; *pCoA*: *p*-coumaric acid; *p*CoFP: *p*-Coumaroyl-feruloylputrescine; QA: Quinic acid; RA: Rosmarinic acid; SA C: Salvianolic acid C; SA E/B/L: Salvianolic acid E/B/L; SA I/H: Salvianolic acid I/H; SF: Salviaflaside; tFA: trans-ferulic acid; TFA: Triferulic acid; TPC: Total Polyphenol Content; Try: Tryptophan.
